# Commentary: Predictive Value of Preoperative Multidetector-Row Computed Tomography for Axillary Lymph Nodes Metastasis in Patients with Breast Cancer

**DOI:** 10.3389/fonc.2021.621967

**Published:** 2021-03-25

**Authors:** Julie Cox, Jonathan Spratt

**Affiliations:** Department of Clinical Radiology, South Tyneside and Sunderland NHS Foundation Trust, Sunderland, United Kingdom

**Keywords:** CT scanning < imaging and diagnostics, breast (diagnostic), axillary node, breast cancer, sentinel lymph biopsy

## Introduction

It is now clear from studies such as the ACOSOG Z0011 randomized controlled trial that a subset group of patients presenting with primary breast cancer with positive (involved) axillary lymph nodes at sentinel lymph node biopsy (SLNB) may be able to avoid an axillary lymph node dissection (ALND) with its associated morbidities (1).

In a retrospective cohort study, Bilimoria (2) reported on a series of 97,314 patients. Twenty-three percent (23%) of patients with SLNB macrometastases and 36% of patients with SNB micrometastases did not have ALND. Yet, for both groups with small volume nodal disease, the axillary local recurrence rate and 5-year survival were unchanged from the group who had undergone ALND, the more extensive operation.

The most rigorous evidence for a stratified approach comes from the ACOSOG Z0011 trial, a prospective randomized controlled trial in which 813 patients with SLNB histology results indicating axillary lymph node involved with metastatic disease, were randomized to ALND *versus* no further surgery.

Additional involved nodes were found in 27% of patients who had ALND. Still, after 6 years of follow-up, there was no significant difference between the two arms of the trial (ALND and no ALND) in terms of local, regional, or overall loco-regional recurrences, or in disease-free survival. Further studies including the POSNOC study (3), a multicenter randomized controlled trial, are currently addressing the same topic or similar topics, although, given the prolonged length of follow-up needed to accurately assess local recurrence in particular, it may be 7 to 8 years before the meaningful results are published after the conclusion of the studies.

As there is an inexorable trend toward less radical surgery in the axilla in the management of patients presenting with breast cancer, the preoperative staging of the axilla is becoming of greater significance. While ultrasound scanning with needle biopsy of some sort (either fine-needle aspiration cytology or wide-bore needle core biopsy) remains the standard of practice internationally, it should be remembered that neither sampling technique has been evaluated context of a rigorous randomized controlled trial for this indication. It could be argued there is a limited evidence base for our current normal practice of preoperative axillary staging.

It is likely that clinical questions about surgery involving the axilla will not be restricted by a binary solution of a positive/negative result, but rather will involve an assessment of the overall burden of disease, as it may well be that low volume axillary nodal disease, found at SLNB, may be managed conservatively.

Multidetector CT has many advantages over ultrasound as an imaging tool. CT provides images in multiplanar images and gives an excellent overview of the anatomy of the axilla, the number of total nodes present, the internal morphology of the nodes, and the proximity of the nodal disease to vascular structures.

Currently, there is no international consensus as to which breast cancer patients should have staging investigation and which specific investigations are indicated. Certainly, there is no agreement that all patients diagnosed with breast cancer should have a staging CT examination.

European (ESMO) guidelines recommend performing chest, and abdominal imaging (US, CT, or MRI scan) and with consideration of a bone scan for patients with clinically positive axillary nodes, large tumors (T3/4), or tumors with aggressive biology (4).

In the UK, the Royal College of Radiologists recommends staging with CT of the chest abdomen and pelvis for patients with large (T4) tumors or with heavy lymph node burden (N2 disease) with or without bone scan and a PET-CT for suspected inflammatory breast cancer (5).

The North American National Comprehensive Cancer Network (NCCN) guidelines recommend a bone scan, abdominal CT/MRI (including the pelvis if symptomatic), chest CT/18F-NaF PET-CT, in symptomatic patients or in stage I-IIB breast cancer with abnormal liver function tests, elevated serum alkaline phosphatase, and localized bone pain (6).

Chen et al. (7) have demonstrated in their cohort of 148 cases of patients with primary breast cancer undergoing axillary surgery that cortical thickness of more than 3 mm and a non-fatty hilum were independent predictors for metastases in the axillary nodes.

As has been demonstrated by other authors, high quality and reproducible images can be achieved by using a simple, standardized CT protocol can be supplied to experienced radiographers, even with CT scanners made by a variety of manufacturers. The intra-operator variability which hinders ultrasound assessment and also the variability of ultrasound equipment performance is therefore avoided. CT images can be reconstructed in any plan of choice for the purposes for example of MDT review or preoperative planning (8).

Multidetector CT has potential in evaluating disease burden before axillary conservation and in follow-up of the “conserved” axilla. Further research using the latest generation of CT scanners is needed to evaluate the effectiveness of CT in this, a different paradigm of radical surgery to the axilla.

## Author Contributions

JC and JS have jointly coauthored this commentary. All authors contributed to the article and approved the submitted version.

## Conflict of Interest

The authors declare that the research was conducted in the absence of any commercial or financial relationships that could be construed as a potential conflict of interest.
